# Integration of Transcriptomic and Single-Cell Data to Uncover Senescence- and Ferroptosis-Associated Biomarkers in Sepsis

**DOI:** 10.3390/biomedicines13040942

**Published:** 2025-04-11

**Authors:** Xiangqian Zhang, Yiran Zhou, Hang Li, Mengru Chen, Fang Peng, Ning Li

**Affiliations:** 1 Department of Blood Transfusion, Clinical Transfusion Research Center, Xiangya Hospital, Central South University, Changsha 410008, China; 2 Department of Clinical Laboratory, Xiangya Hospital, Central South University, Changsha 410008, China; 3 National Clinical Research Center for Geriatric Disorders, Xiangya Hospital, Central South University, Changsha 410008, China; 4 Department of Internal Medicine IV (Gastroenterology, Hepatology, and Infectious Diseases), Jena University Hospital, 07743 Jena, Germany; 5 NHC Key Laboratory of Cancer Proteomics, Xiangya Hospital, Central South University, Changsha 410008, China; 6 Department of Oncology, Xiangya Hospital, Central South University, Changsha 410008, China

**Keywords:** sepsis, senescence, ferroptosis

## Abstract

**Background:** Sepsis is a life-threatening condition characterized by organ dysfunction due to an imbalanced immune response to infection, with high mortality. Ferroptosis, an iron-dependent cell death process, and cellular senescence, which exacerbates inflammation, have recently been implicated in sepsis pathophysiology. **Methods:** Weighted gene co-expression network analysis (WGCNA) was used to identify ferroptosis- and senescence-related gene modules in sepsis. Differentially expressed genes (DEGs) were analyzed using public datasets (GSE57065, GSE65682, and GSE26378). Receiver operating characteristic (ROC) analysis was performed to evaluate their diagnostic potential, while single-cell RNA sequencing (scRNA-seq) was used to assess their immune-cell-specific expression. Molecular docking was conducted to predict drug interactions with key proteins. **Results:** Five key genes (*CD82*, *MAPK14*, *NEDD4*, *TXN*, and *WIPI1*) were significantly upregulated in sepsis patients and highly correlated with immune cell infiltration. *MAPK14* and *TXN* exhibited strong diagnostic potential (AUC = 0.983, 0.978). Molecular docking suggested potential therapeutic interactions with diclofenac, flurbiprofen, and N-acetyl-L-cysteine. **Conclusions:** This study highlights ferroptosis and senescence as critical mechanisms in sepsis and identifies promising biomarkers for diagnosis and targeted therapy. Future studies should focus on clinical validation and precision medicine applications.

## 1. Introduction

Sepsis is characterized by life-threatening organ dysfunction resulting from an imbalanced host response to infection [1,2]. Estimates indicate that, between 1990 and 2017, approximately 48.9 million people were affected by sepsis globally, with approximately 11 million deaths, accounting for 19.7% of all fatalities worldwide [3,4]. As the leading cause of death in intensive care units (ICUs), sepsis places a heavy burden on the global economy and public health systems because of its high prevalence and lethality [5,6]. The pathogenesis is complex and varied, encompassing multiple pathological processes, such as systemic inflammatory responses, immune dysregulation, and circulatory and microcirculatory dysfunction, which are intertwined and ultimately lead to organ failure and metabolic disorders [7,8]. Therefore, sepsis has become a highly fatal clinical emergency that urgently requires rapid identification and effective interventions to improve patient prognosis [9]. Therefore, an in-depth analysis of the underlying molecular mechanisms of sepsis is crucial not only for developing scientific and effective preventive strategies but also for providing a solid theoretical basis for promoting the innovation of therapeutic approaches and the optimization of clinical management.

Ferroptosis and senescence have emerged as key mechanisms of interest in the pathological process of sepsis [10,11]. As an iron-dependent mode of cell death, ferroptosis is deeply embedded in the complex pathological network of the inflammatory response, oxidative stress, and lipid peroxidation injury and plays a central role in sepsis-induced organ dysfunction [12,13]. Sepsis essentially results from an imbalance between infection-induced inflammation and the anti-inflammatory system [2,7]. Iron, a key mediator of bacterial infection, is deeply involved in the metabolism of pathogenic bacteria and exacerbates the deterioration caused by tissue damage by promoting the production of fatty acids and reactive oxygen species (ROS) [14,15]. Drugs such as dexmedetomidine and irisin have been shown to effectively attenuate sepsis-related injury by modulating ferroptosis-associated molecules, suggesting that targeting ferroptosis holds promising therapeutic potential in the management of sepsis [16,17].

Recent studies have identified ferroptosis as a critical process in the progression of inflammation, potentially bridging the regulatory gap between inflammation and senescence [18]. ROS are important mediators in the induction of inflammation and senescence, and cellular ferroptosis is an important source of ROS and is positively correlated with inflammation progression [19,20,21]. Serum ferritin and stored iron levels increase significantly with age, and iron-ion accumulation is particularly pronounced in certain tissues of elderly individuals [22]. This phenomenon is closely linked to a variety of senescence-related diseases, including neurodegenerative conditions, where the role of ferroptosis has been extensively documented [23]. Furthermore, iron chelators show great promise in modulating inflammation and oxidative stress, as well as in addressing senescence-related processes, paving the way for innovative therapeutic strategies in the future [24,25]. These findings highlight the critical need for further investigations into ferroptosis and senescence as therapeutic targets, offering the potential for innovative treatments that could significantly improve the management and prognosis of sepsis patients.

However, the relationship between ferroptosis and senescence remains complex and contradictory. On the one hand, senescence leads to impaired cellular metabolic function, reduced ferritin degradation, and diminished iron excretion, resulting in the accumulation of iron ions in senescent cells [26]. On the other hand, senescent cells exhibit significant resistance to ferroptosis, hindering their clearance and leading to their continued accumulation and dysfunction [27]. This paradox highlights that ferroptosis and senescence are not only independent risk factors for sepsis but also form a tightly interconnected pathological network through complex interactions.

By systematically analyzing the specific mechanisms of ferroptosis and senescence in sepsis and developing targeted intervention strategies to precisely regulate these pathological processes, it may be possible to significantly improve the prognosis of sepsis patients. The concept of the synergistic regulation of ferroptosis and senescence provides a novel perspective for sepsis treatment and management. Within the framework of personalized and precision medicine, research on the interplay between ferroptosis and senescence is expected to become a key focus area, potentially leading to advancements in sepsis treatment modalities and providing mechanistic insights and practical guidance for advancing medical science.

In this study, we utilized weighted gene co-expression network analysis (WGCNA) to systematically investigate the core genes associated with ferroptosis and senescence in sepsis, aiming to identify novel diagnostic and therapeutic targets and address critical gaps in this field. Notably, we explored the pivotal roles of these core genes in immune cell infiltration and, for the first time, systematically integrated them with single-cell RNA sequencing data for in-depth analysis. This novel research approach not only offers a fresh perspective on the pathogenesis of sepsis but also provides a preliminary theoretical foundation for developing targeted diagnostic and therapeutic strategies. By further advancing our understanding of sepsis, this study offers potential insights into the field, which may contribute to improvements in clinical diagnosis and treatment outcomes.

## 2. Materials and Methods

### 2.1. Data Preprocessing and Identification of Key Genes Associated with Ferroptosis and Senescence

To identify key genes related to ferroptosis and senescence, two publicly available transcriptomic datasets, GSE57065 (25 normal samples, 82 sepsis samples) and (GSE6568242 normal controls, 760 patients with sepsis), were retrieved from the Gene Expression Omnibus (GEO) database. The samples in the above datasets are from blood samples. Data normalization and log2 transformation were performed using the limma package (version 3.60.6) in R [28]. Batch effects in the two datasets were corrected using the ComBat algorithm from the sva package (version 3.52.0) [29]. The effectiveness of normalization and batch correction was assessed using principal component analysis (PCA). Subsequently, differentially expressed genes (DEGs) between the sepsis and control groups were identified from the merged dataset. The criteria for defining DEGs were |log2 fold change (logFC)| > 0.6 and adjusted *p*-value < 0.05. Heatmaps were generated using the pheatmap package (version 1.0.12) to visualize expression differences, and volcano plots were created with the ggplot2 package (version 3.5.1) to illustrate significant DEGs [30]. Gene sets related to ferroptosis and senescence were obtained from the MSigDB, and the specific genes are listed in Table S1 of Supplementary File S1.

### 2.2. Construction of Co-Expression Networks and Identification of Sepsis-Related Modules via WGCNA

To further explore gene–gene interactions and identify sepsis-related modules, weighted gene co-expression network analysis (WGCNA) was conducted on the GSE57065 and GSE65682 datasets using the WGCNA package (version 1.73) in R [31]. For each dataset, the optimal soft-thresholding power was selected based on the scale-free topology criterion. Adjacency matrices were then computed and transformed into topological overlap matrices (TOMs), which describe network connectivity. Hierarchical clustering was applied to TOM-based dissimilarity measures, and modules were defined by dynamic tree cutting. The resulting modules were correlated with clinical traits (sepsis vs. control) using Pearson correlation analysis. Module–trait relationships were visualized through dendrograms, module color plots, and correlation heatmaps. Genes from significantly correlated modules were retained for downstream integration with DEGs and functional gene sets. To characterize the biological functions of sepsis-associated modules, genes within these modules were subjected to Reactome pathway enrichment analysis using the ReactomePA package (version 1.48.0) [32]. To determine shared genes of interest, a Venn diagram was constructed using the VennDiagram package (version 1.7.3) in R to illustrate the overlap among the DEGs [33], ferroptosis-related genes, senescence-related genes, and genes from WGCNA modules. To further explore the mechanism of action of the intersecting genes, we subjected the intersecting genes obtained from the above analysis to GO-KEGG analysis.

### 2.3. Validation of Differential Expression of Key Genes Across Datasets

The expression levels of the intersecting genes in the merged dataset (GSE57065 and GSE65682) were analyzed and further validated via an independent dataset (GSE26378). GSE26378 contains expression data for whole-blood samples from 82 children with septic shock and 21 normal controls. The datasets were preprocessed via the limma package (version 3.60.6) for normalization and batch correction in the merged dataset to ensure comparability [28]. Violin plots were generated via the ggpubr package (version 0.6.0) to visualize differences in gene expression between the control and sepsis groups in both datasets [34]. Statistical significance was assessed via the Wilcoxon rank-sum test, with a threshold of *p* < 0.05 indicating significant differential expression.

### 2.4. Evaluation of the Diagnostic Performance of Key Genes via ROC Curve Analysis

To evaluate the diagnostic performance of the intersecting genes, receiver operating characteristic (ROC) curve analysis was conducted via the pROC package (version 1.18.5) on both the merged dataset (GSE57065 and GSE65682) and an independent validation dataset (GSE26378) [35]. GSE26378 contains expression data from whole-blood samples from 82 children with septic shock and 21 normal control children. For the merged dataset, batch correction was performed prior to analysis to ensure data consistency. ROC curves were generated to assess the sensitivity and specificity of each gene in distinguishing the sepsis group from the control group. The area under the curve (AUC) and 95% confidence interval (CI) were calculated to quantify the diagnostic accuracy via the robust functionality of the pROC package (version 1.18.5) [35]. Similar analyses were repeated for the validation dataset to confirm the reproducibility of the findings. The results provide a comprehensive assessment of the diagnostic potential of these intersecting genes.

### 2.5. Development and Evaluation of a Diagnostic Prediction Model for Sepsis

After the datasets GSE57065 and GSE65682 were integrated, a diagnostic prediction model was developed via multivariate logistic regression and the rms package (version 6.8.2) in R software(version 4.4.1) [36], incorporating the expression levels of the intersecting genes and clinical information as predictors and clinical diagnosis (control or sepsis) as the outcome. A nomogram was constructed to visualize the contributions of individual genes and to estimate the total risk score for diagnosis. Model performance was evaluated via three metrics: (1) a calibration curve to assess the agreement between the predicted probabilities and actual outcomes via the rms package [36]; (2) a receiver operating characteristic (ROC) curve, which was calculated with the pROC package (version 1.18.5) [35], to quantify the model’s discriminative ability via the area under the curve (AUC); and (3) decision curve analysis (DCA), which was used to evaluate the clinical utility of the model across varying risk thresholds. The calibration curve and DCA are supported by the rms and rmda R packages (version 4.4.3). Bootstrapping with 1000 repetitions was conducted to validate the robustness of the model.

### 2.6. Analyzing the Association Between Key Genes and Immune Cell Infiltration via Bioinformatics

To investigate the relationships between the intersecting genes and immune cell infiltration, correlation analyses were conducted using immune cell composition data derived from CIBERSORT. The merged dataset (GSE57065 and GSE65682) was utilized to estimate immune cell proportions, and Spearman’s correlation coefficients were calculated to assess the associations between gene expression levels and immune cell fractions. The analyses were visualized via a heatmap and a network plot, both generated with the ggplot2 and linkET packages (version 0.0.7.4), illustrating the correlations of all five genes with various immune cells. The correlation results are displayed in bar plots and included in the results and Supplementary Materials. Statistical significance was set at *p* < 0.05.

### 2.7. Comprehensive Analysis of scRNA-Seq Data to Identify Gene Expression Patterns in Sepsis

Single-cell RNA sequencing (scRNA-seq) data from patients with advanced sepsis and healthy individuals were obtained from the GEO dataset GSE175453, which includes whole-blood samples from four septic patients and five controls. Quality control was performed by removing cells with fewer than 200 genes, more than 7000 genes, or mitochondrial gene content greater than 20%. The data were then log-normalized. Highly variable genes were identified based on the variance-to-mean expression ratio. Principal component analysis (PCA) was used for dimensionality reduction, followed by clustering using the Louvain algorithm and visualization with t-distributed stochastic neighbor embedding (t-SNE). To annotate cell types, we employed the SingleR (version 2.6.0) package, referencing the Human Primary Cell Atlas, which maps clusters to known immune cell types using canonical marker genes. Additionally, we confirmed marker gene expression patterns within each cluster, ensuring consistency between assigned identities and transcriptional signatures. Expression levels of the five intersecting genes were visualized in t-SNE plots and summarized in a heatmap to demonstrate their distribution across immune cell subsets. The key R packages utilized in the analysis included Seurat for preprocessing (version 5.2.1), normalization, clustering, and visualization of scRNA-seq data; ggplot2 (version 3.5.1) for generating t-SNE plots and heatmaps [30]; SingleR (version 2.6.0) for annotating cell clusters [37]; and harmony (version 1.2.3) for batch correction and SCpubr (version 2.0.2) for creating publication-ready visualizations [38,39].

### 2.8. Molecular Docking to Explore Ligand–Protein Interactions

Molecular docking was performed to evaluate the binding interactions of the ligands with proteins. The protein structures were retrieved from the Protein Data Bank (PDB), and the ligand structures were obtained from PubChem or constructed via ChemDraw (version 19.0). Protein preparation involved removing water molecules and adding polar hydrogens via AutoDock Tools (version 1.5.7). Ligand preparation included energy minimization to achieve optimal conformations. Docking simulations were conducted via AutoDock Vina (version 1.2.5), with the docking grid centered around the active site of each protein. The binding affinities (kcal/mol) were calculated, and the binding modes were visualized via PyMOL (version 2.5) to identify interactions such as hydrogen bonding, hydrophobic contacts, and electrostatic interactions.

## 3. Results

### 3.1. Key Genes Linking Ferroptosis and Senescence Identified Through Bioinformatics Analysis in Sepsis

**Figure 1:**
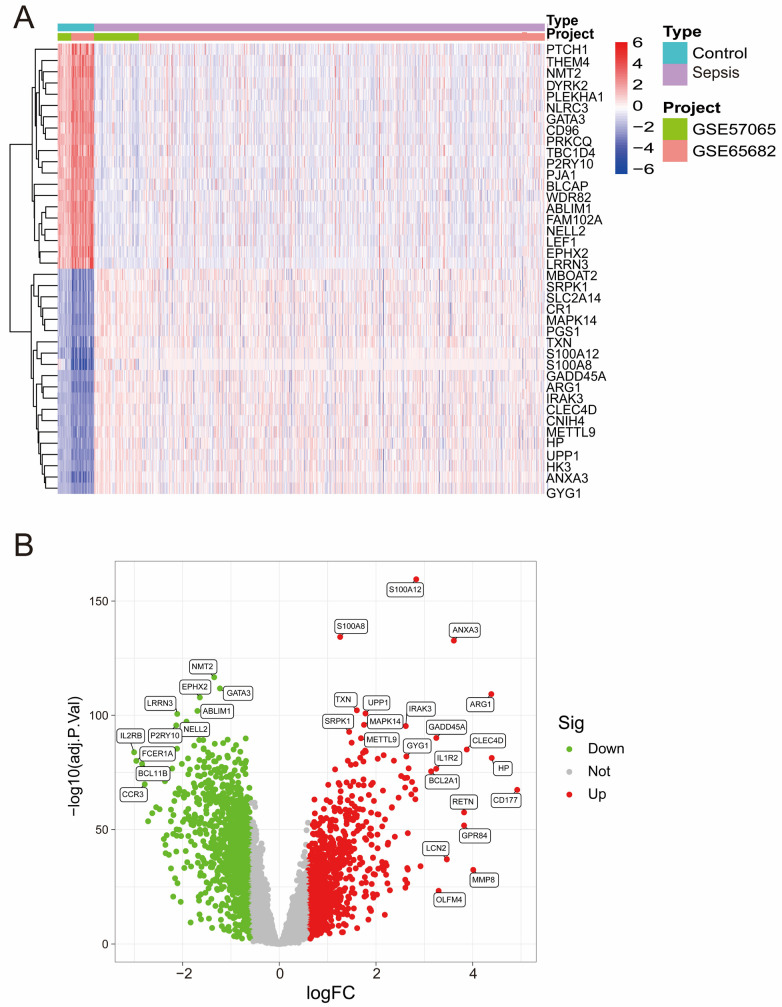
(**A**) A heatmap of differentially expressed genes (DEGs) across the merged dataset. The rows represent genes, and the columns represent samples. Red and blue indicate high and low expression levels, respectively. (**B**) Volcano plot of DEGs from the merged dataset. Significantly upregulated (red) and downregulated (green) genes are shown (|logFC| > 0.6, adjusted *p*-value < 0.05).

After batch correction (Figure S1A,B), PCA demonstrated improved consistency between the GSE57065 and GSE65682 datasets, confirming the successful removal of batch effects. Heatmap analysis (Figure 1A) highlighted distinct expression profiles of DEGs across the merged dataset, revealing a clear separation between the control and sepsis groups. Differential expression analysis revealed several significant DEGs, as shown in the volcano plot (Figure 1B), with upregulated genes (red) and downregulated genes (green) meeting the threshold criteria.

### 3.2. Identification of Sepsis-Associated Co-Expression Modules

**Figure 2:**
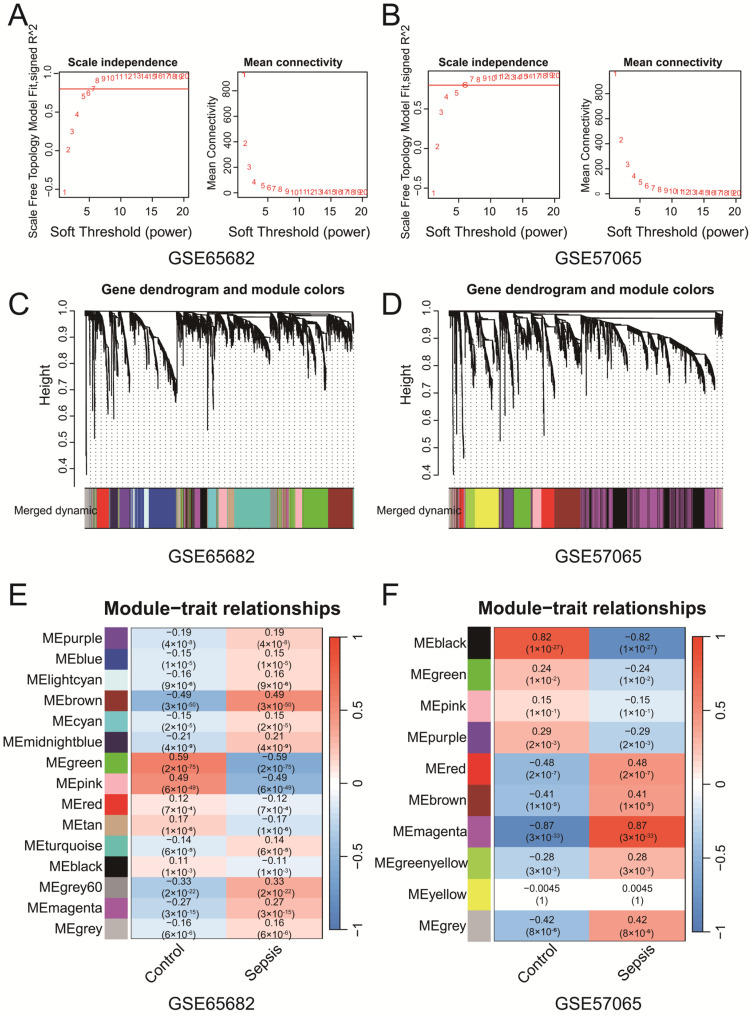
(**A**,**B**) The selection of soft-thresholding powers for GSE65682 (**A**) and GSE57065 (**B**). The selected powers (7 for (**A**), 6 for (**B**)) ensured scale-free topology with sufficient mean connectivity. (**C**,**D**) Gene clustering dendrograms and module assignments for GSE65682 (**C**) and GSE57065 (**D**). Modules are represented by distinct colors, and similar modules were merged on the basis of eigengene similarity. (**E**,**F**) Heatmaps of module–trait correlations for GSE65682 (**E**) and GSE57065 (**F**). Each cell shows the Pearson correlation coefficient and *p*-value.

WGCNA of GSE65682 and GSE57065 identified key gene co-expression modules associated with clinical traits. Soft-thresholding power selection (Figure 2A,B) determined the optimal power to be 7 for GSE65682 and 6 for GSE57065, ensuring scale-free topology with sufficient mean connectivity. Gene clustering dendrograms (Figure 2C,D) revealed distinct co-expression modules, represented by unique colors, which were refined through merging on the basis of eigengene similarity. Module–trait correlation heatmaps (Figure 2E,F) revealed significant associations between modules and clinical traits. In the GSE65682 dataset, the brown module demonstrated a significant negative correlation with the control group (r = −0.49, *p* = 3 × 10^−50^) and a significant positive correlation with the sepsis group (r = 0.49, *p* = 3 × 10^−50^). Similarly, in the GSE57065 dataset, the magenta module presented the strongest positive correlation with the sepsis group (r = 0.87, *p* < 3 × 10^−33^). These findings suggest that the brown and magenta modules are more strongly associated with their respective clinical conditions, highlighting that specific co-expressed modules are significantly associated with sepsis. The genes identified from each module of GSE57065 and GSE65682 have been uploaded as part of Supplementary File S2, with subsequent analyses primarily focusing on the brown and magenta modules. The Venn diagram (Figure S1C) revealed five intersecting genes (*CD82*, *MAPK14*, *NEDD4*, *TXN*, and *WIPI1*) among ferroptosis-related genes, senescence-related genes, WGCNA-identified modules from both datasets and the DEGs from the merged dataset (Merge-Diff). These genes represent potential key regulators linking ferroptosis, senescence, and sepsis. In addition, we performed Reactome pathway enrichment analysis on the brown module from the WGCNA results of GSE65682 and the magenta module from GSE57065. The analysis revealed several pathways associated with immune and inflammatory responses. Detailed results are provided in Supplementary File S1 Table S2. In addition, we found that the above five genes are associated with multiple ferroptosis and senescence pathways, as shown in Supplementary File S1 Table S3.

### 3.3. Ferroptosis- and Senescence-Associated Genes Are Significantly Upregulated in Sepsis Samples

**Figure 3:**
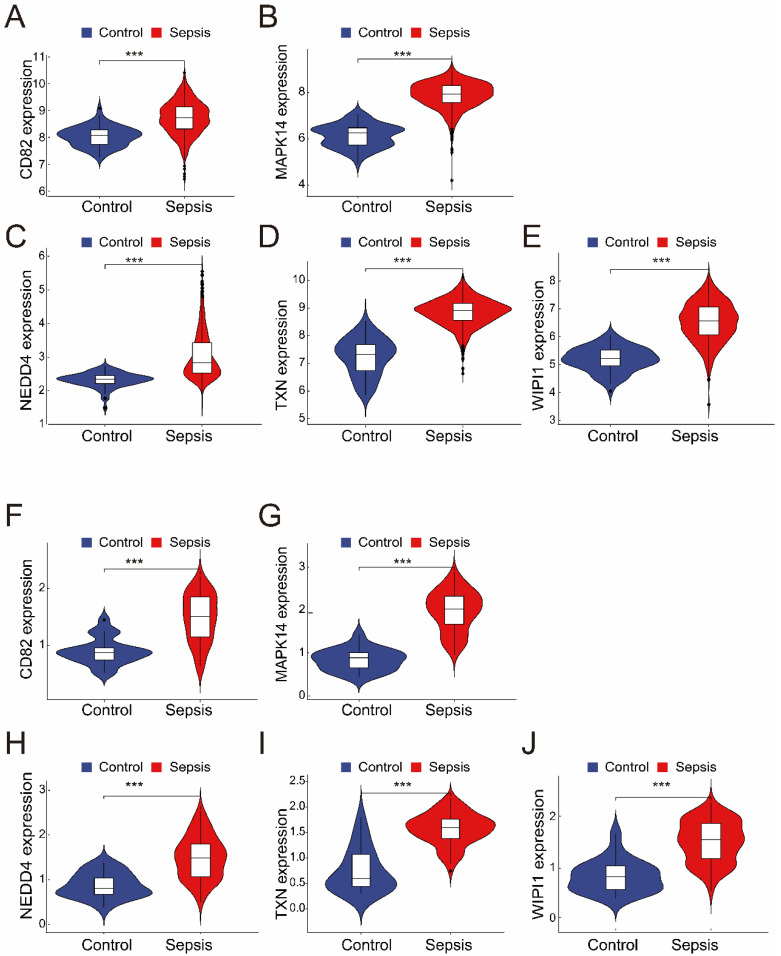
(**A**–**E**) The expression of the five intersecting genes (*CD82*, *MAPK14*, *NEDD4*, *TXN*, and *WIPI1*) in the merged dataset (GSE57065 and GSE65682). Violin plots revealed significantly greater expression levels in the sepsis group than in the control group (*** *p* < 0.001). (**F**–**J**) Validation of the five genes in the independent dataset (GSE26378). Like in the merged dataset, all five genes (*CD82*, *MAPK14*, *NEDD4*, *TXN*, and *WIPI1*) were significantly upregulated in the sepsis group (*** *p* < 0.001).

The merged dataset (GSE57065 and GSE65682) revealed that all five genes (*CD82*, *MAPK14*, *NEDD4*, *TXN*, and *WIPI1*) were significantly upregulated in the sepsis group compared with the control group (Figure 3A–E). These findings were consistent with those from the validation dataset (GSE26378), where the expression levels of the same five genes were also significantly greater in the sepsis group (Figure 3F–J). These results highlight the robustness and consistency of the identified gene expression patterns, suggesting their potential as reliable biomarkers for sepsis diagnosis and as therapeutic targets.

### 3.4. Ferroptosis- and Senescence-Related Genes Exhibit Strong Diagnostic Predictive Power in Sepsis Samples

**Figure 4:**
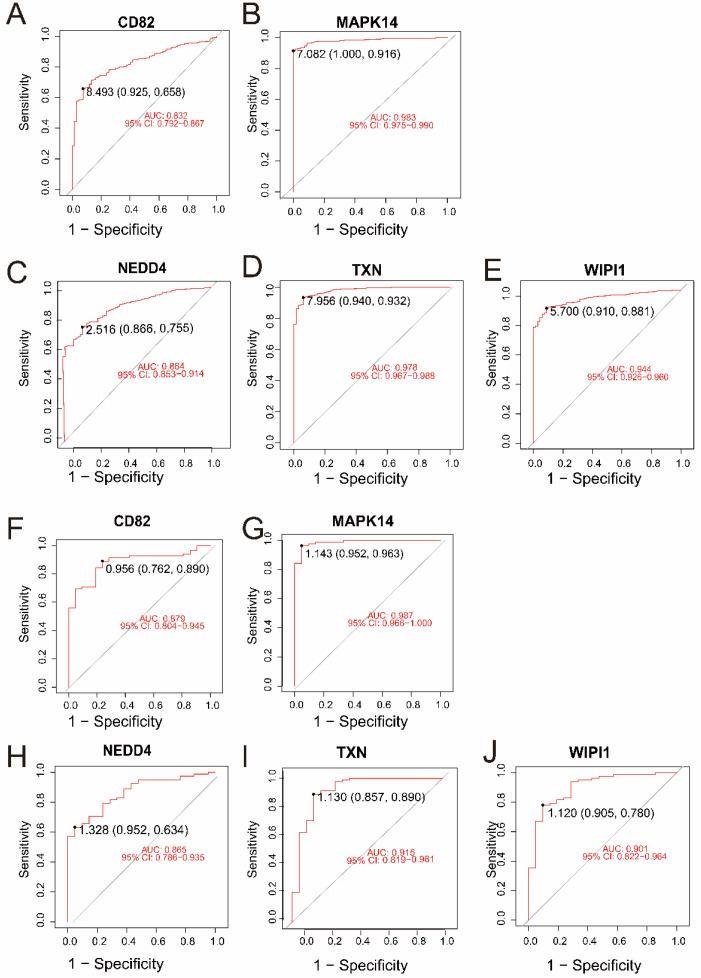
(**A**–**E**) ROC curves for the five intersecting genes (*CD82*, *MAPK14*, *NEDD4*, *TXN*, and *WIPI1*) in the merged dataset (GSE57065 and GSE65682). The AUC values and 95% confidence intervals indicate excellent diagnostic accuracy for all genes. (**F**–**J**) ROC curves for the same five genes in the validation dataset (GSE26378). Similar to the merged dataset, all genes exhibited high diagnostic performance, with MAPK14 and TXN achieving the highest AUC values.

In the merged dataset (GSE57065 and GSE65682), all five genes—*CD82*, *MAPK14*, *NEDD4*, *TXN*, and *WIPI1*—demonstrated excellent diagnostic performance, with AUC values ranging from 0.832 to 0.983 (Figure 4A–E). Among them, *MAPK14* (AUC = 0.983, 95% CI: 0.975–0.990) and *TXN* (AUC = 0.978, 95% CI: 0.967–0.988) exhibited the highest diagnostic accuracy. Validation in an independent dataset (GSE26378) further confirmed the strong diagnostic potential of these genes, with AUC values ranging from 0.865 to 0.987 (Figure 4F–J). Consistently, *MAPK14* (AUC = 0.987, 95% CI: 0.966–1.000) and *TXN* (AUC = 0.915, 95% CI: 0.819–0.981) showed robust diagnostic ability. These results highlight the reliability of *CD82*, *MAPK14*, *NEDD4*, *TXN*, and *WIPI1* as potential biomarkers for sepsis and control samples.

### 3.5. Outstanding Predictive Accuracy and Clinical Utility of the Diagnostic Model in Sepsis Samples

**Figure 5:**
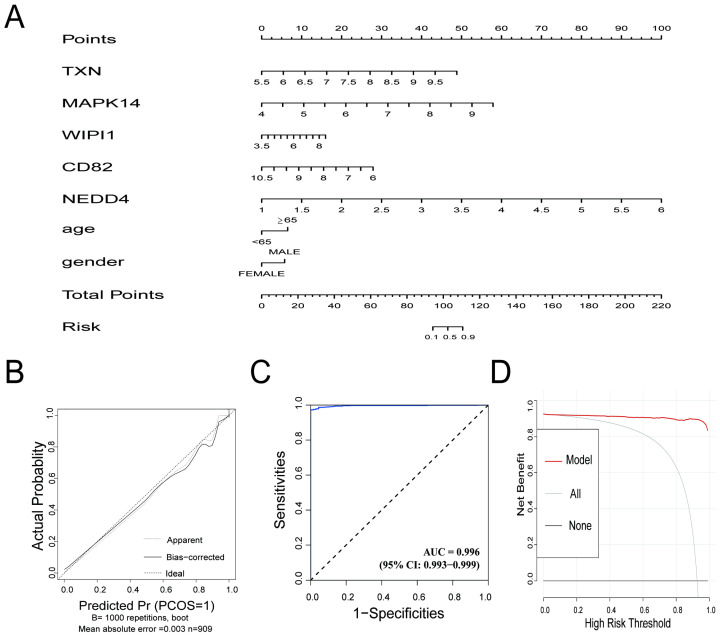
(**A**) Nomogram for the diagnostic prediction model. The contribution of each gene (*TXN*, *MAPK14*, *WIPI1*, *CD82*, and *NEDD4*) and clinical information are represented, and the total points correspond to the predicted risk of sepsis. (**B**) Calibration curve for the prediction model. The apparent line (dotted) and bias-corrected line (solid) show the agreement between the predicted probabilities and actual outcomes. The ideal line represents perfect calibration. (**C**) Receiver operating characteristic (ROC) curve for the prediction model. The AUC value of 0.996 demonstrates excellent discriminative performance. (**D**) Decision curve analysis (DCA) showing the net benefit of the prediction model across different high-risk thresholds. The red line (model) outperforms the “all” (gray) and “none” (black) strategies, highlighting the model’s clinical utility.

The diagnostic prediction model demonstrated excellent predictive performance for distinguishing between the control and sepsis groups. The nomogram (Figure 5A) illustrates the individual contributions of the five genes (*TXN*, *MAPK14*, *WIPI1*, *CD82*, and *NEDD4*) and clinical information (age, gender) to the overall risk score, providing a practical tool for estimating the probability of sepsis. The calibration curve (Figure 5B) indicated strong agreement between the predicted probabilities and actual outcomes, with a mean absolute error of 0.003. The ROC curve (Figure 5C) showed exceptional discriminative ability, with an AUC of 0.996 (95% CI: 0.993–0.999), reflecting high sensitivity and specificity. Decision curve analysis (Figure 5D) demonstrated that the model provided a significant net benefit across a wide range of risk thresholds, confirming its potential clinical applicability. The confusion matrix, presented in Supplementary File S1 Table S4, highlights the model’s strong classification performance. Sensitivity reached 99.29%, and precision was 98.93%. The model also achieved an overall accuracy of 98.35% and an F1 score of 0.9911, underscoring its robustness in correctly identifying true positive cases while minimizing false positives. These results highlight the utility of the model for accurate and clinically relevant diagnostics in integrated datasets.

### 3.6. Key Genes Are Significantly Correlated with Immune Cell Infiltration in Sepsis Samples

**Figure 6:**
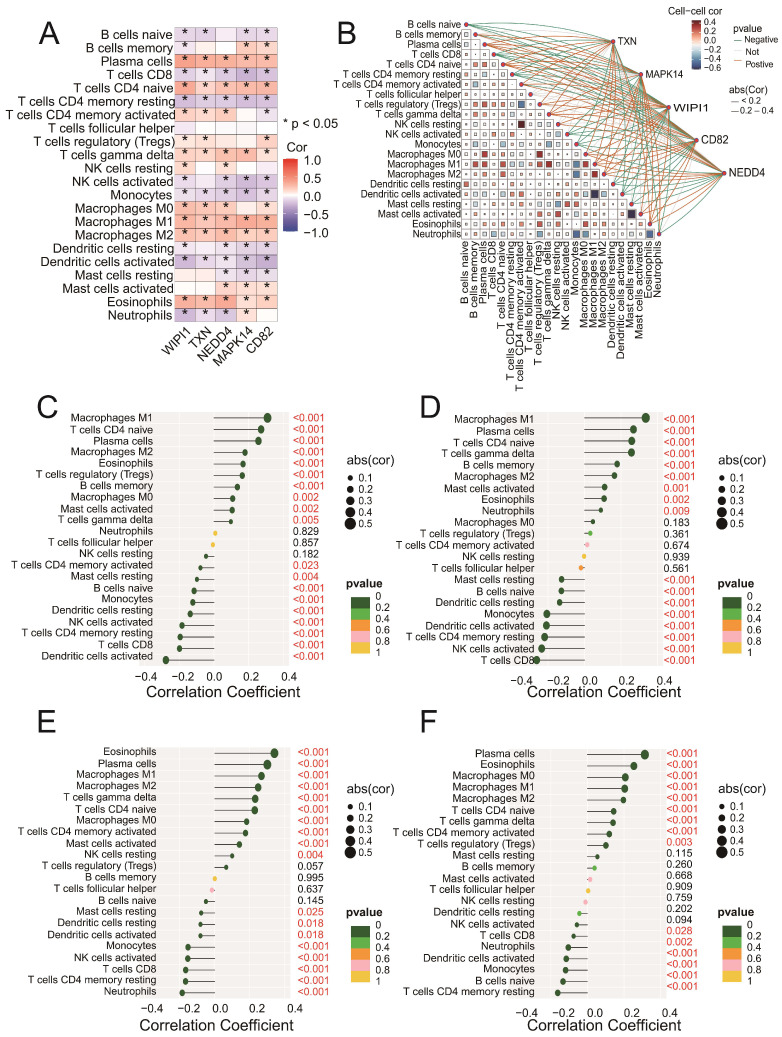
(**A**) A heatmap showing the Spearman correlation coefficients between the expression levels of the five intersecting genes (*CD82*, *MAPK14*, *NEDD4*, *TXN*, and *WIPI1*) and immune cell fractions. Positive correlations are indicated in blue, negative correlations are in red, and statistically significant correlations are marked with asterisks (* *p* < 0.05). (**B**) A network plot illustrating the interactions between immune cells and the five intersecting genes. The blue edges represent positive correlations, whereas the red edges indicate negative correlations. The edge thickness reflects the strength of the correlation. (**C**–**F**) Lollipop plots illustrating the correlation coefficients between *CD82* (**C**), *MAPK14* (**D**), *NEDD4* (**E**), and *TXN* (**F**) and immune cell fractions derived from the CIBERSORT analysis. The x-axis represents the correlation coefficients, whereas the y-axis represents the immune cell types. Each dot represents the strength of the correlation for a specific immune cell type, with the corresponding bars extending to the x-axis.

Correlation analysis revealed that all five genes (*CD82*, *MAPK14*, *NEDD4*, *TXN*, and *WIPI1*) were significantly associated with multiple immune cell types (Figure 6A). Among these, positive correlations were observed with M1 macrophages and plasma cells, whereas negative correlations were found with CD8 T cells and regulatory T cells (Tregs). The network plot (Figure 6B) highlights the interactions between immune cells and genes, showing both positive (blue edges) and negative (red edges) correlations. Specific analyses of *CD82* (Figure 6C), *MAPK14* (Figure 6D), *NEDD4* (Figure 6E), and *TXN* (Figure 6F) revealed significant associations with immune cell subsets, including M1 macrophages, activated memory CD4 T cells, and eosinophils. These findings suggest that the intersecting genes may play roles in regulating immune responses. Detailed correlation data for WIPI1 can be found in Figure S1D. Together, these results emphasize the intricate involvement of key genes in modulating immune cell dynamics and highlight their potential as targets for therapeutic intervention in sepsis.

### 3.7. Distinct Immune Cell Clusters and Gene Expression Profiles Highlight Cellular Diversity in Sepsis Samples

**Figure 7:**
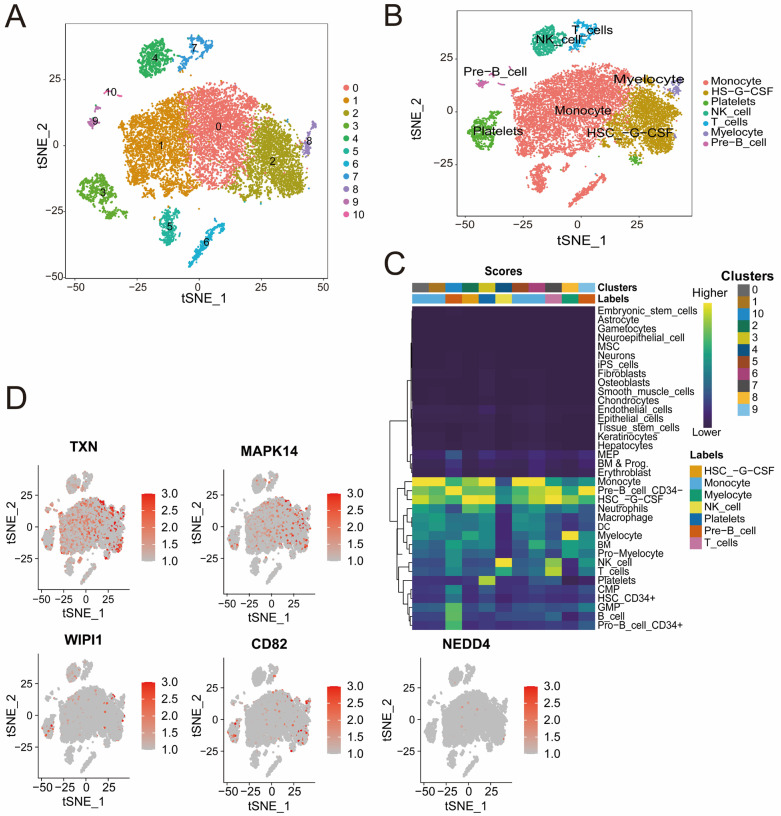
(**A**,**B**) t-SNE plots illustrating cell clustering (**A**) and cell type annotation on the basis of canonical markers (**B**). The colors represent distinct clusters, and the labels indicate the cell types. (**C**) Heatmap showing the distribution of cell clusters and their associated cell types identified through single-cell RNA sequencing analysis. The color intensity represents the score of each cluster corresponding to specific cell types, including NK cells, B cells, monocyte cells, and others. (**D**) t-SNE plots displaying the expression patterns of *CD82*, *MAPK14*, *NEDD4*, *TXN*, and *WIPI1.* Red indicates increased expression, and gray indicates decreased expression.

Quality control analysis revealed a clear separation of high-quality cells on the basis of RNA count and mitochondrial gene content. Highly variable genes were identified, highlighting the robustness of the dataset for clustering (Figure S1E,F). The t-SNE cluster analysis identified 11 distinct cell populations that were further divided into seven subpopulations, including monocytes, T cells, NK cells, and pre-B cells, all of which were annotated according to the identified marker genes (Figure 7A,B). The heatmap (Figure 7C) shows the distribution of cell clusters and their associated cell types from single-cell RNA sequencing analysis. The intensity reflects cluster scores for specific cell types, such as T cells, NK cells, and monocyte cells, highlighting the diversity and composition of cell populations during sepsis. The cluster-specific expression patterns of five genes (*CD82*, *MAPK14*, *NEDD4*, *TXN*, and *WIPI1*) were visualized, revealing high expression levels in specific immune cell subpopulations (Figure 7D). For example, *TXN* and *MAPK14* are highly expressed in monocytes, whereas *WIPI1*, *CD82*, and *NEDD4* are expressed at varying levels across T cells and B cells. These findings offer valuable insights into the cellular heterogeneity of sepsis and underscore the potential for identifying novel therapeutic targets to address the complex immune dysregulation observed in this condition.

### 3.8. Potential Binding of Diclofenac, Flurbiprofen, and N-Acetyl-L-Cysteine to Key Proteins

**Figure 8:**
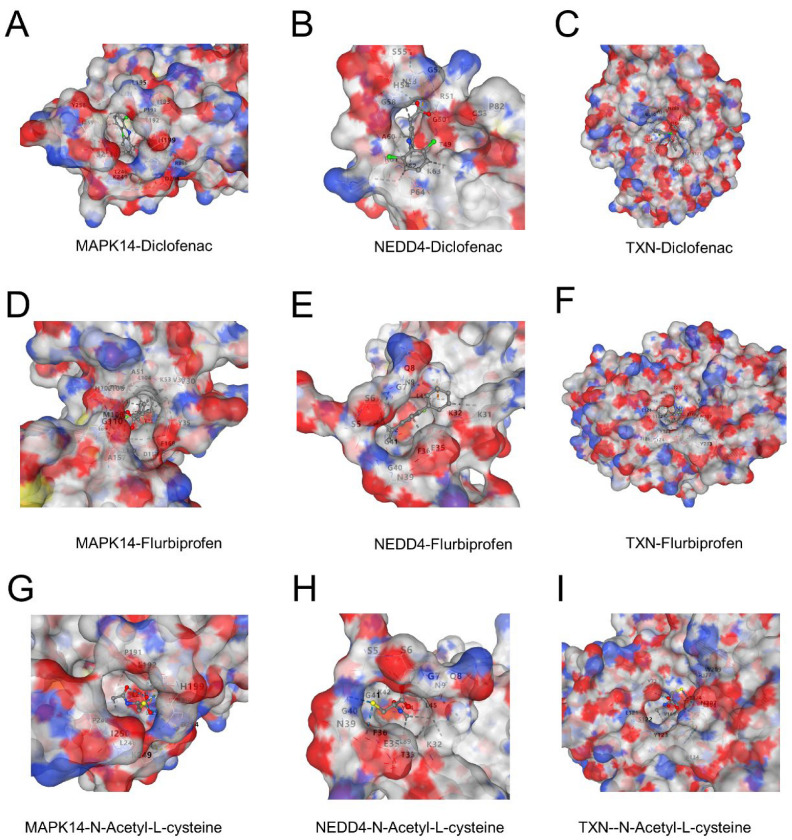
(**A**–**C**) Binding interactions of diclofenac with MAPK14 (**A**), NEDD4 (**B**), and TXN (**C**). The surface representation highlights the ligand docked in the active site, showing key hydrophobic and electrostatic interactions. (**D**–**F**) Binding interactions of flurbiprofen with MAPK14 (**D**), NEDD4 (**E**), and TXN (**F**). The ligand forms stable interactions in the active sites of the proteins, with notable hydrogen bonds in NEDD4. (**G**–**I**) Binding interactions of N-acetyl-L-cysteine with MAPK14 (**G**), NEDD4 (**H**), and TXN (**I**). The interactions are dominated by hydrogen bonding, contributing to moderate binding affinities.

Molecular docking revealed favorable binding interactions between three ligands (diclofenac, flurbiprofen, and N-acetyl-L-cysteine) and the proteins MAPK14, NEDD4, and TXN. Diclofenac exhibited strong binding affinities with all three proteins, forming hydrophobic and electrostatic interactions (Figure 8A–C). Flurbiprofen showed comparable binding energy with distinctive interactions in the active sites of the proteins, particularly in MAPK14 and NEDD4 (Figure 8D–F). N-Acetyl-L-cysteine demonstrated moderate binding affinity, with interactions primarily mediated by hydrogen bonds, indicating potential therapeutic relevance (Figure 8G–I). These findings suggest that the three ligands may target these proteins effectively and could provide a basis for further experimental validation.

## 4. Discussion

Sepsis, a systemic inflammatory syndrome induced by infection-induced immune dysregulation, is characterized by persistent infection, excessive inflammation, and immunosuppression, which often leads to organ dysfunction, posing a serious threat to life. It has become one of the key triggers leading to death in ICUs [40,41]. Ferroptosis and cellular senescence have been shown to play critical roles in the pathophysiology of sepsis [42,43]. Ferroptosis is driven primarily by inflammation-induced redox imbalance, leading to the inhibition of the cystine/glutamate antiporter, reduced glutathione synthesis, and elevated lipid peroxidase activity [13]. This cascade triggers lipid peroxidation of cell membranes, resulting in cell death and exacerbating organ damage. Similarly, under the influence of sepsis-induced inflammation, senescent cells release SASP factors such as IL-6 and TNF-α [44]. These factors form a positive feedback loop that amplifies inflammation, recruits immune cells, intensifies the inflammatory storm, and secretes matrix metalloproteinases, which degrade the extracellular matrix and hinder tissue repair [45]. The synergistic effects of ferroptosis and cellular senescence contribute to the progression of sepsis and its poor prognosis; therefore, an in-depth investigation of these two mechanisms provides important guidelines for the search for novel therapeutic targets.

In this study, we utilized the GEO datasets GSE57065 and GSE65682 to analyze gene expression profiles in sepsis patients and normal controls. DEGs were identified and subsequently intersected with genes related to ferroptosis and senescence, as were the results of WGCNA derived from the different datasets. This approach led to the identification of five key genes: *CD82*, *MAPK14*, *NEDD4*, *TXN*, and *WIPI1*. To further investigate the potential biological functions of the five key genes, we performed GO and KEGG enrichment analyses. The results revealed that these genes were significantly enriched in several critical biological pathways, including cellular senescence, regulation of autophagy [46,47], oxidative stress response [48,49], response to radiation [50,51], and lipid oxidation [52,53]. These findings further support the important roles of these genes in the regulation of cellular senescence and ferroptosis. In the combined dataset, all five genes tended to be significantly upregulated in the sepsis group compared with the control group. This trend was further validated in the GSE26378 dataset, confirming the robust differential expression of these genes in sepsis samples. Additionally, ROC curve analysis revealed that the AUC values for all five genes exceeded 0.8 in both the combined and validation datasets, indicating strong predictive ability. Among them, *MAPK14* demonstrated the highest predictive performance for sepsis (AUC = 0.983), suggesting its potential as a sensitive marker for detecting sepsis-related changes and its involvement in the underlying mechanisms of the disease. These findings provide a solid foundation for further exploration of sepsis pathogenesis.

*MAPK14*, a member of the MAPK family, plays a critical role in the response to stress signals such as inflammatory stimuli and oxidative stress. By activating downstream signaling pathways, it mediates essential cellular responses, underscoring its importance in regulating physiological and pathological processes [54]. *MAPK14* is highly expressed in the myocardial and lung tissues of septic mice [55]. Further, *MAPK14* has been reported to have considerable value in the early diagnosis of sepsis in children [56]. Celastrol inhibits the activation of the MAPK- and PI3K/AKT-related pathways; reduces the inflammatory response, apoptosis, and oxidative stress; and significantly ameliorates LPS-induced acute hepatic injury [57]. Fangji Fuling Decoction (FFD) can ameliorate the LPS-induced inflammatory response in septic mice by inhibiting the MAPK14/FOXO3A signaling pathway [58]. *Mapk14*, a potential sepsis-related positive regulator gene, may contribute to sepsis through ferroptosis and senescence. CD82 is a transmembrane protein that is considered a metastatic oncogene and plays a key role in cancer recurrence [59]. CD82 may regulate the balance of intracellular ROS production and clearance by interacting with other membrane proteins. The interaction between CD82 and DARC triggers an intracellular signaling cascade and induces tumor cell senescence [60]. *CD82* has been reported to inhibit phagocyte migration, but support macrophage activation, and *CD82*-positive macrophages play a role in inflammatory regression [61]. *TXN* encodes thioredoxin, which is a critical antioxidant protein that mitigates oxidative stress by regulating cellular free radicals and ROS. Its role in preventing cell death and senescence has been widely documented. Research indicates that elevated *TXN* expression in conditions such as Parkinson’s disease and cancer impedes the progression of ferroptosis [62,63]. The silencing of TXN and an imbalance in Trx induce cellular senescence [64,65]. By reducing oxidative stress and stabilizing iron metabolism proteins, TXN upregulation preserves iron homeostasis, potentially halting oxidative-damage-induced senescence and ferroptosis. This process is vital for maintaining organ function and mitigating sepsis-induced multiorgan dysfunction. The *NEDD4* gene, which is part of the NEDD4 family, encodes an E3 ubiquitin ligase. Studies have revealed that its deficiency exacerbates sepsis and cell death in mice [66]. *WIPI1* contributes to autophagosome assembly and binds phosphoinositides, which are essential components of any membrane [67]. It also facilitates transferrin receptor recycling to the plasma membrane, underscoring its role in intracellular trafficking and membrane dynamics [68]. These findings highlight the critical involvement of these key genes in sepsis pathophysiology, suggesting that targeting *MAPK14*, *CD82*, *TXN*, *NEDD4*, and *WIPI1* may offer promising therapeutic strategies for modulating ferroptosis, senescence, and immune responses to improve patient outcomes in sepsis.

In this study, a diagnostic prediction model integrating five key genes (*TXN*, *MAPK14*, *WIPI1*, *CD82*, and *NEDD4*) and clinical information (age, gender) was innovatively constructed. In contrast to traditional diagnostic methods, this model can accurately capture expression changes in multiple key genes by virtue of its unique design and thus predict the diagnosis of sepsis, greatly improving the accuracy and timeliness of early diagnosis. This innovative approach significantly shortens the window period of traditional diagnostic methods and creates more possibilities for timely medical interventions, thus laying a solid foundation for improving patient prognosis.

To further explore the mechanism of action of these genes in sepsis, the five identified ferroptosis- and senescence-related genes were analyzed to determine whether they were significantly different and significantly correlated with immune cells. Strong positive correlations were observed with M1 macrophages, whereas negative correlations were found with CD8+ T cells and Tregs. Furthermore, as shown in Figure 6C–E, *CD82*, *MAPK14*, *NEDD4*, and *TXN* were significantly positively correlated with M1-type macrophage infiltration, which is consistent with previous reviews indicating that M1-type macrophages are a key factor in the intense inflammatory response and tissue damage in the early stages of sepsis-induced acute lung injury [69]. M1-like macrophages continue to increase in number and release large amounts of inflammatory factors that cause severe inflammatory responses, such as IL-1, TNF-α, and IL-6 [70,71]. Tregs are a group of cells with immunosuppressive functions that suppress inflammation, mainly by secreting immunosuppressive molecules [72]. The important role of Treg cells in sepsis has been reported in various studies [73]. These findings underscore the intricate relationships among ferroptosis, senescence, and immune regulation in sepsis, revealing that the modulation of key genes may offer novel therapeutic targets for controlling inflammation and immune dysfunction, ultimately improving sepsis management and patient outcomes.

To explore the cellular and molecular characteristics of immune cell subsets involved in sepsis, we utilized the publicly available scRNA-seq dataset GSE175453. This analysis identified seven immune cell subpopulations within the PBMCs of sepsis patients. The expression of five key genes was assessed across these immune cell clusters in sepsis samples, and the results reveal their pivotal roles in inducing functional changes in immune cells during sepsis. For example, *TXN* and *MAPK14* are expressed predominantly in monocytes, whereas *WIPI1*, *CD82*, and *NEDD4* exhibit variable expression in T and B cells, highlighting the diversity and composition of immune populations during sepsis.

To further evaluate the possibility of the above five genes as potential therapeutic targets for sepsis, we carried out molecular docking analyses. Due to the lack of clear protein structural information for CD82 and WIPI1, the three target genes with resolvable structures, MAPK14, TXN, and NEDD4, were finally selected for docking studies. The results show that three drugs—diclofenac, flurbiprofen, and N-acetyl-L-cysteine—can all bind to the above target proteins. Previous reports indicate that diclofenac, a widely used non-steroidal anti-inflammatory drug, exerts its anti-inflammatory and analgesic effects mainly by inhibiting the activity of the cyclooxygenases COX-1 and COX-2, thereby reducing prostaglandin synthesis [74]. Currently, it is mainly used for the treatment of rheumatic diseases and inflammation associated with the musculoskeletal system [75]. In addition, evidence suggests that diclofenac may be involved in cellular senescence by inhibiting telomerase activity and inducing cycle arrest in colorectal cancer cells, accompanied by the upregulation of the expression of the senescence markers p53 and p21 proteins [76]. Flurbiprofen, a phenylpropanoid acid non-steroidal anti-inflammatory drug, also acts mainly by inhibiting COX-2-mediated prostaglandin synthesis and is clinically used in rheumatoid arthritis, degenerative osteoarthropathies, ankylosing spondylitis, and other diseases [77,78]. Previous studies have found that flurbiprofen promotes cellular senescence in a senescence model constructed from human dermal fibroblasts [79]. N-acetyl-L-cysteine, a commonly used antioxidant and mucolytic agent, plays an important role in maintaining cellular redox homeostasis as a precursor of glutathione (GSH) [80]. It is widely regarded as a potent scavenger of reactive oxygen species (ROS) with ferroptosis-inhibitory activity [81,82]. N-acetyl-L-cysteine has also been shown to slow down the process of cellular senescence [83,84] and has demonstrated organ-protective effects in a sepsis model [85]. Taken together, diclofenac, flurbiprofen, and N-acetyl-L-cysteine may regulate ferroptosis and cellular-senescence-related pathways through specific binding to MAPK14, TXN, and NEDD4, thus providing potential pharmacological intervention strategies for sepsis treatment.

Although this study provides novel insights into the mechanisms of sepsis and identifies potential diagnostic and therapeutic targets, certain limitations remain. The analysis was based solely on GEO datasets with relatively small sample sizes and lacked experimental and clinical validation.

Despite these limitations, our findings offer strong theoretical support for understanding the roles of ferroptosis and cellular senescence in sepsis. The identified key genes and the diagnostic model lay the groundwork for the future development of early detection tools and targeted therapies. Further studies incorporating larger clinical cohorts and experimental verification are warranted to confirm the clinical utility and mechanistic relevance of these findings.

## Data Availability

The data and R scripts utilized in this study can be obtained by contacting the corresponding author.

## Supplementary Materials

The following supporting information can be downloaded at: https://www.mdpi.com/article/10.3390/biomedicines13040942/s1, Figure S1; Supplementary File S1; Supplementary File S2.

## Abbreviations

AUC	area under the curve
CI	confidence interval
DEGs	differentially expressed genes
DCA	decision curve analysis
Merge-Diff	DEGs from the merged dataset
ICUs	intensive care units
PCA	principal component analysis
PDB	Protein Data Bank
ROC	receiver operating characteristic
ROS	reactive oxygen species
SASP	senescence-associated secretory phenotype
scRNA-seq	single-cell RNA sequencing
t-SNE	t-distributed stochastic neighbor embedding
Tregs	regulatory T cells
WGCNA	weighted gene expression network analysis
