# Supplementary material for: Integration of Transcriptomic and Single-Cell Data to Uncover Senescence- and Ferroptosis-Associated Biomarkers in Sepsis
Source: Biomedicines. 2025 Apr 11;13(4):942. doi: 10.3390/biomedicines13040942 (PMC12025025; doi:10.3390/biomedicines13040942)
Supplement: Supplementary file 1 [file biomedicines-13-00942-s001.zip › Figure S1.pdf]

Figure S1

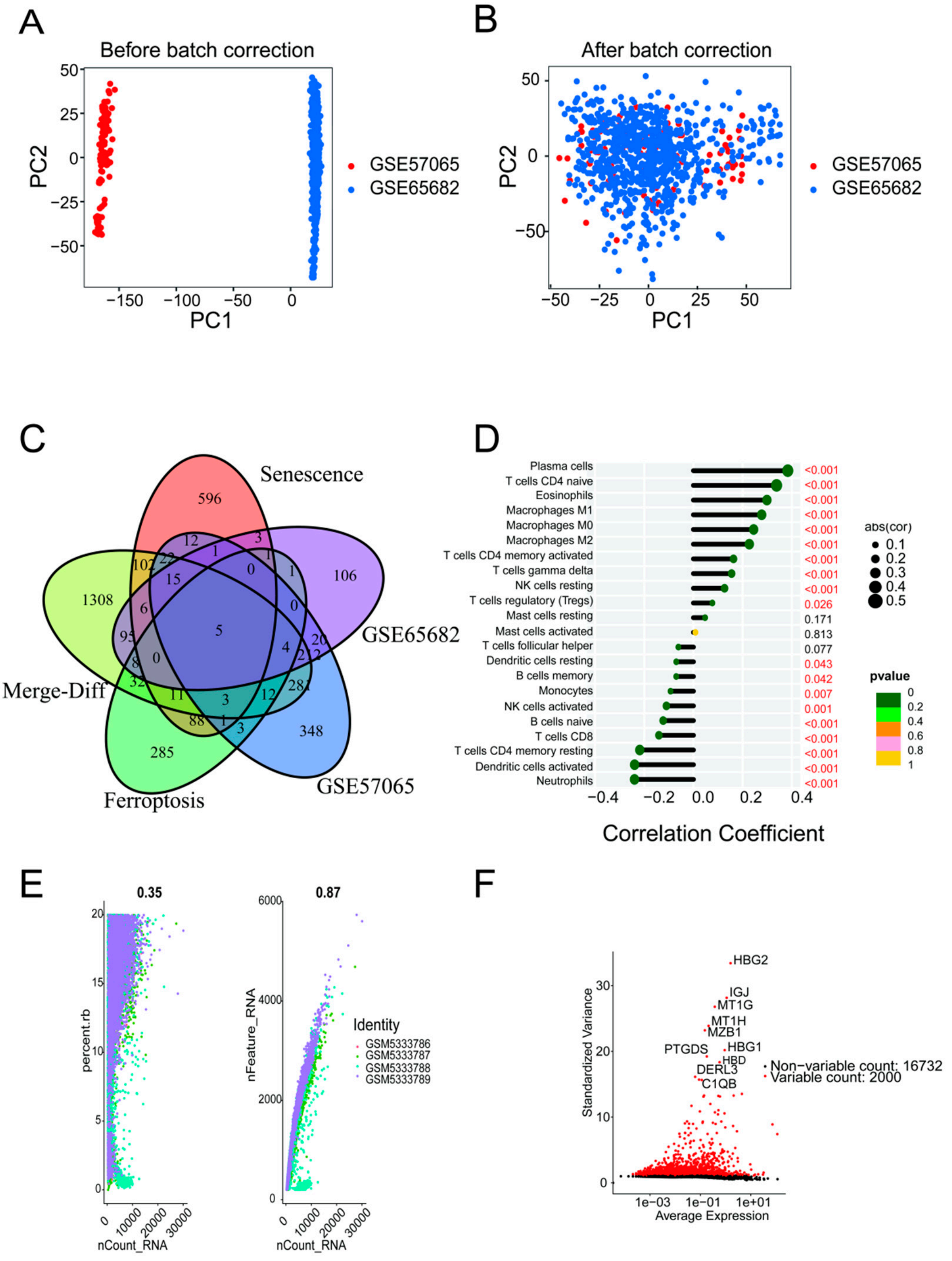

Figure S1 A-B: Principal component analysis (PCA) before (A) and after (B) batch correction for the GSE57065 and GSE65682 datasets. Batch correction aligns the datasets, improving consistency for downstream analysis. **C**: Venn diagram showing the overlap of DEGs from the merged dataset (Merge-Diff), ferroptosis-related genes, senescence-related genes, and WGCNA-identified modules from GSE57065 and GSE65682. The intersection identified five shared genes: *CD82*, *MAPK14*, *NEDD4*, *TXN*, and *WIPI1*. **D:** Lollipop plots illustrating the correlation coefficients between WIPI1 and immune cell fractions derived from CIBERSORT analysis. The x-axis represents the correlation coefficients, whereas the y-axis represents the immune cell types. Each dot represents the strength

of the correlation for a specific immune cell type, with the corresponding bars extending to the x-axis. **E**: Quality control of the scRNA-seq data. The left plot shows the RNA count (nCount_RNA), and the right plot shows mitochondrial gene expression across cells. The filtering thresholds are indicated. **F**: Identification of highly variable genes. The scatterplot displays the mean expression (x-axis) and variability (y-axis) of genes. Variable genes are highlighted in red.
